# Editorial for the Special Issue on Integrated Photonics and Optoelectronics

**DOI:** 10.3390/mi15091090

**Published:** 2024-08-29

**Authors:** He Yang, Xinyang Su

**Affiliations:** 1 School of Instrumentation and Optoelectronic Engineering, Beihang University, Beijing 100191, China; 2 School of Physical Science and Engineering, Beijing Jiaotong University, Beijing 100044, China

Integrated photonic and optoelectronic technologies have become powerful tools in the drive to develop devices that are much smaller and more highly integrated, with lower power consumption and higher functionality. The trend in research and development (R&D) towards the miniaturization of optical and optoelectrical devices and systems [1,2,3,4,5], similarly to recent interest in microelectronics [6,7], has led to a continuous research interest in various integrated photonic and optoelectronic structures and devices [8,9,10,11]. Thus, various easy-to-integrate nanomaterials [12,13,14,15,16,17] with excellent optical and electrical properties, as well as high-precision nanofabrication techniques, have been developed in the last two decades, which have witnessed tremendous progress in integrated photonic and optoelectronic devices.

In this Special Issue (SI) on Integrated Photonics and Optoelectronics, we include 15 papers focusing on the latest research advances both in physical theory and device applications. Through the publication of these results, we hope to present the latest research developments in current challenges and future opportunities for the advancement of integrated photonic and optoelectronic devices, in order to promote the application of these devices in our daily life. Specifically, in this SI, we discuss three current topics in the field of light–matter interactions, including the fundamental theory, such as surface plasma, metasurfaces, and the active manipulation of the optical fields. Twelve publications are included that discuss the latest research on integrated photonic and optoelectronic devices, such as organic light-emitting devices, edge-emitting diode lasers, liquid crystal–quantum dot hybrids, a miniature Fourier transform spectrometer, rod fiber picosecond amplifiers, a thin-film integrated optical intraocular pressure sensor, polarization beam splitters (PBSs), hollow-core anti-resonant fibers, and Mach–Zehnder interferometer (MZI)-based matrix computation.

In particular, Liu et al. [18] numerically demonstrated and investigated a compact and high-power THz radiation source based on the excitation of the surface plasmon polaritons (SSP) mode on a roofed metallic grating by an electron beam. Wang et al. [19] increased the light extraction efficiency of organic light-emitting devices using electrochemically corroded patterned substrates. Shen et al. [20] demonstrated a double-strip array-based metasurface that supports the sharp quasi-bound states in the continuum (quasi-BICs) in terahertz regions. Han et al. [21] introduced the progress of edge-emitting diode lasers, based on a coupled-waveguide concept. Xi et al. [22] investigated an all-optical controlled THz modulator based on a Bi_2_Te_3_/Si heterostructure under 532 nm and 405 nm continuous-wave (CW) laser illumination. Schmelz et al. [23] presented and evaluated a possible fabrication process based on colloidal polystyrene (PS) nanosphere lithography for the fabrication of such anti-resonant (AR) structures on arbitrarily shaped fused silica substrates. Sun et al. [24] studied several of the most traditional hollow-core anti-resonant fiber (HC-ARF) structures, with the aim of achieving low confinement loss, single-mode performance, and a high insensitivity to bending in the 2 µm band. Bezrukov et al. [25] reported on the developing approaches to modulate the optical behavior of the microfluidic devices, which is conducted by infusing smart hybrids of liquid crystal and quantum dots into microchannel confinement. Zhang et al. [26] proposed a miniature Fourier transform spectrometer using a thin-film lithium niobate electro-optical modulator, instead of the conventional modulator made by titanium diffusion in lithium niobate. Liu et al. [27] enhanced the coupling efficiency of the picosecond rod-type fiber amplifier by carefully setting the optimal mode–field matching. Xu et al. [28] designed a thin-film integrated optical intraocular pressure sensor based on the interferometry principle, which could read out the intraocular pressure value from interference patterns and monitor the value changes in real time simultaneously. Kotlyar et al. [29] discussed the superposition of several parallel identical Laguerre–Gaussian beams with single rings. Sun et al. [30] investigated the spin thermal radiation in a twisted bilayer α-MoO3 metasurface. Mei et al. [31] proposed and realized a PBS based on surface plasmonic resonance in a designed photonic crystal fiber (PCF). Hou et al. [32] identified the main hardware error sources of MZI-based matrix computation, summarized the available hardware error correction methods from the perspective of the entire MZI meshes and a single MZI device, and proposed a new architecture that will largely improve the precision of MZI-based matrix computations without increasing the size of the MZI’s mesh.

## References

[1] Pérez D., Gasulla I., Das Mahapatra P., Capmany J. (2020). Principles, fundamentals, and applications of programmable integrated photonics. Adv. Opt. Photonics.

[2] Wang X., Cui Y., Li T., Lei M., Li J., Wei Z. (2018). Recent Advances in the Functional 2D Photonic and Optoelectronic Devices. Adv. Opt. Mater..

[3] Liang G., Yu X., Hu X., Qiang B., Wang C., Wang Q.J. (2021). Mid-infrared photonics and optoelectronics in 2D materials. Mater. Today.

[4] Sinatkas G., Christopoulos T., Tsilipakos O., Kriezis E.E. (2021). Electro-optic modulation in integrated photonics. J. Appl. Phys..

[5] You J., Luo Y., Yang J., Zhang J., Yin K., Wei K., Zheng X., Jiang T. (2020). Hybrid/Integrated Silicon Photonics Based on 2D Materials in Optical Communication Nanosystems. Laser Photonics Rev..

[6] Yang L., Hu H., Scholz A., Feist F., Cadilha Marques G., Kraus S., Bojanowski N.M., Blasco E., Barner-Kowollik C., Aghassi-Hagmann J. (2023). Laser printed microelectronics. Nat. Commun..

[7] Dieny B., Prejbeanu I.L., Garello K., Gambardella P., Freitas P., Lehndorff R., Raberg W., Ebels U., Demokritov S.O., Akerman J. (2020). Opportunities and challenges for spintronics in the microelectronics industry. Nat. Electron..

[8] Liu C., Yan X., Song X., Ding S., Zhang D.W., Zhou P. (2018). A semi-floating gate memory based on van der Waals heterostructures for quasi-non-volatile applications. Nat. Nanotechnol..

[9] Zhang Z., Chen P., Duan X., Zang K., Luo J., Duan X. (2017). Robust epitaxial growth of two-dimensional heterostructures, multiheterostructures, and superlattices. Science.

[10] Sena-Torralba A., Alvarez-Diduk R., Parolo C., Piper A., Merkoci A. (2022). Toward Next Generation Lateral Flow Assays: Integration of Nanomaterials. Chem. Rev..

[11] Li W., Wang C., Lu X. (2021). Integrated transition metal and compounds with carbon nanomaterials for electrochemical water splitting. J. Mater. Chem. A.

[12] Elbanna A., Jiang H., Fu Q., Zhu J.F., Liu Y., Zhao M., Liu D., Lai S., Chua X.W., Pan J. (2023). 2D Material Infrared Photonics and Plasmonics. ACS Nano.

[13] Cheng Z., Cao R., Wei K., Yao Y., Liu X., Kang J., Dong J., Shi Z., Zhang H., Zhang X. (2021). 2D Materials Enabled Next-Generation Integrated Optoelectronics: From Fabrication to Applications. Adv. Sci..

[14] Singh A., Jo S.S., Li Y., Wu C., Li M., Jaramillo R. (2020). Refractive Uses of Layered and Two-Dimensional Materials for Integrated Photonics. ACS Photonics.

[15] Huang Y., Sutter E., Shi N., Zheng J., Yang T., Englund D., Gao H., Sutter P. (2015). Reliable exfoliation of large-area high-quality flakes of graphene and other two-dimensional materials. ACS Nano.

[16] Lee H.Y., Kim S. (2022). Nanowires for 2D material-based photonic and optoelectronic devices. Nanophotonics.

[17] Yan C., Gan L., Zhou X., Guo J., Huang W., Huang J., Jin B., Xiong J., Zhai T., Li Y. (2017). Space-Confined Chemical Vapor Deposition Synthesis of Ultrathin HfS2 Flakes for Optoelectronic Application. Adv. Funct. Mater..

[18] Liu Y., Zhang X., Wang Y., Cai H., Sun J., Zhu Y., Li L. (2024). Excitation of Terahertz Spoof Surface Plasmons on a Roofed Metallic Grating by an Electron Beam. Micromachines.

[19] Wang Y., Li Z., Bai Y., Wang Y. (2024). Increasing the Light Extraction Efficiency of Organic Light-Emitting Devices by Electrochemically Corroded Patterned Substrates. Micromachines.

[20] Shen Y., Wang J., Sheng H., Li X., Yang J., Liu H., Liu D. (2024). Double-Strip Array-Based Metasurfaces with BICs for Terahertz Thin Membrane Detection. Micromachines.

[21] Han L., Wang Z., Gordeev N.Y., Maximov M.V., Tang X., Beckman A.A., Kornyshov G.O., Payusov A.S., Shernyakov Y.M., Zhukov A.E. (2023). Progress of Edge-Emitting Diode Lasers Based on Coupled-Waveguide Concept. Micromachines.

[22] Xi Y., Zhou Y., Cao X., Wang J., Lei Z., Lu C., Wu D., Shi M., Huang Y., Xu X. (2023). Broadband All-Optical THz Modulator Based on Bi_2_Te_3_/Si Heterostructure Driven by UV-Visible Light. Micromachines.

[23] Schmelz D., Jia G., Käsebier T., Plentz J., Zeitner U.D. (2023). Antireflection Structures for VIS and NIR on Arbitrarily Shaped Fused Silica Substrates with Colloidal Polystyrene Nanosphere Lithography. Micromachines.

[24] Sun T., Su X., Meng F., Wang Z., Song J., Zhang C., Xu T., Zhang Y., Zhang H., Cui M. (2023). Design of 2 μm Low-Loss Hollow-Core Anti-Resonant Fibers. Micromachines.

[25] Bezrukov A., Galyametdinov Y. (2023). Dynamic Flow Control over Optical Properties of Liquid Crystal–Quantum Dot Hybrids in Microfluidic Devices. Micromachines.

[26] Zhang L., Gou G., Chen J., Li W., Ma W., Li R., An J., Wang Y., Liu Y., Yan W. (2023). Miniature Fourier Transform Spectrometer Based on Thin-Film Lithium Niobate. Micromachines.

[27] Liu D., Mao X., Bi G., Li T., Zang D., Sun N. (2023). Efficiency Enhancing Technique for Rod Fiber Picosecond Amplifiers with Optimal Mode Field Matching. Micromachines.

[28] Xu X., Liu Z., Wang L., Huang Y., Yang H. (2023). High−Accuracy Film−Integrated Optical Sensor for Real−Time Intraocular Pressure Monitoring. Micromachines.

[29] Kotlyar V.V., Kovalev A.A., Kozlova E.S., Savelyeva A.A. (2022). Tailoring the Topological Charge of a Superposition of Identical Parallel Laguerre–Gaussian Beams. Micromachines.

[30] Sun Y., Zhang D., Wu B., Liu H., Yang B., Wu X. (2022). Metasurfaces Assisted Twisted α-MoO_3_ for Spinning Thermal Radiation. Micromachines.

[31] Mei C., Wu Y., Yuan J., Qiu S., Zhou X. (2022). Design of Compact and Broadband Polarization Beam Splitters Based on Surface Plasmonic Resonance in Photonic Crystal Fibers. Micromachines.

[32] Hou H., Xu P., Zhou Z., Su H. (2023). Hardware Error Correction for MZI-Based Matrix Computation. Micromachines.

